# An open-source human-in-the-loop BCI research framework: method and design

**DOI:** 10.3389/fnhum.2023.1129362

**Published:** 2023-06-27

**Authors:** Martin Gemborn Nilsson, Pex Tufvesson, Frida Heskebeck, Mikael Johansson

**Affiliations:** ^1^Department of Automatic Control, Lund University, Lund, Sweden; ^2^Ericsson Research, Lund, Sweden; ^3^Department of Psychology, Lund University, Lund, Sweden

**Keywords:** brain-computer interface, research framework, online, real-time, EEG

## Abstract

Brain-computer interfaces (BCIs) translate brain activity into digital commands for interaction with the physical world. The technology has great potential in several applied areas, ranging from medical applications to entertainment industry, and creates new conditions for basic research in cognitive neuroscience. The BCIs of today, however, offer only crude online classification of the user's current state of mind, and more sophisticated decoding of mental states depends on time-consuming offline data analysis. The present paper addresses this limitation directly by leveraging a set of improvements to the analytical pipeline to pave the way for the next generation of online BCIs. Specifically, we introduce an open-source research framework that features a modular and customizable hardware-independent design. This framework facilitates human-in-the-loop (HIL) model training and retraining, real-time stimulus control, and enables transfer learning and cloud computing for the online classification of electroencephalography (EEG) data. Stimuli for the subject and diagnostics for the researcher are shown on separate displays using web browser technologies. Messages are sent using the Lab Streaming Layer standard and websockets. Real-time signal processing and classification, as well as training of machine learning models, is facilitated by the open-source Python package Timeflux. The framework runs on Linux, MacOS, and Windows. While online analysis is the main target of the BCI-HIL framework, offline analysis of the EEG data can be performed with Python, MATLAB, and Julia through packages like MNE, EEGLAB, or FieldTrip. The paper describes and discusses desirable properties of a human-in-the-loop BCI research platform. The BCI-HIL framework is released under MIT license with examples at: bci.lu.se/bci-hil (or at: github.com/bci-hil/bci-hil).

## 1. Introduction

**Figure 1:**
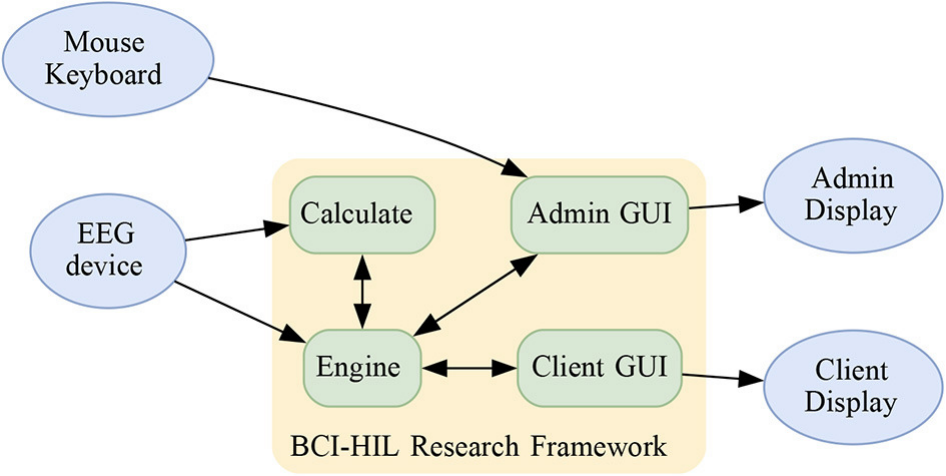
The BCI-HIL research framework separates the software into four major parts: The BCI-HIL research framework separates the software into four major parts. The *Engine* is the main module, keeping track of the current state of the experiment as well as the distribution of messages and data between other parts in BCI-HIL. The Engine also takes care of storing unprocessed EEG data for offline analysis. The *Calculate* module handles EEG preprocessing, machine learning, creating epochs, training, and performing inference. The *Admin GUI* handles commands from the experiment admin and presents the current status and live values in a dashboard. The *Client GUI* presents stimulus for the subject and receives input from the subject.

The ability to accurately decode mental states, including perceptions, thoughts, and emotions in real-time would represent a significant advancement in numerous research fields and provide a wide range of potential applications. A brain-computer interface (BCI) is a device that interprets brain activity to enable direct human-to-machine communication without using regular pathways such as peripheral nerves or muscles (Wolpaw et al., 2002). While brain activity may be measured using a variety of methods such as functional magnetic resonance imaging (fMRI; Belliveau et al., 1991), magnetoencephalography (MEG; Cohen, 1968), and functional near-infrared spectroscopy (fNIRS; Jöbsis, 1977), the examples and discussions presented in this paper are given primarily with non-invasive electroencephalography (EEG; Berger, 1929) in mind. The temporal resolution of the EEG is high and thus well-suited for BCI research and applications. A fundamental limitation with current BCIs is that more advanced decoding is time-consuming and would require offline data analysis. Thus, the next generation of BCIs critically depends on the development of analytical tools to speed up and enhance the online classification of brain data. In this paper, we provide a BCI human-in-the-loop (HIL) framework for this purpose. The main components of BCI-HIL are visualized in Figure 1.

### 1.1. Brief history of BCI research

“Über das Elektrenkephalogramm des Menschen" was written in 1929, 5 years after the first successful recording of the Electroencephalogram by Berger (1929).

In 1973, one of the first brain-computer interface setups was described by Vidal (1973). In the coming years, BCI research expanded to include the development of more sophisticated systems that could be used to assist or augment human cognitive or motor functions.

BCI research is advancing with a focus on the development of more user-friendly and effective BCI systems, by exploring the use of machine learning algorithms to improve the performance and reliability of BCIs, and to enable them to be used in a wider range of applications. The field is continuing to evolve and grow as the potential applications of BCIs expand, leading to new and exciting possibilities for the future.

In order to improve machine learning models, it may be necessary to question the current scientific methodology that has been traditionally used. New deep learning models will obscure certain details, and we will need to approach them as black-box models, where we no longer have the ability to explicitly determine the purpose of individual components. The adoption of deep neural networks may require a holistic perspective, in contrast to the reductionist method of current scientific practice, where machine learning may assume the task of comprehending and generating models. This epistemological shift could potentially lead to new and creative methods in our research toolbox. However, deep neural networks require huge training datasets, which can solely be obtained through yet-to-be-seen ubiquitous BCI consumer products used in everyday life.

### 1.2. Different types of BCI systems

Based on how information is fundamentally passed from the brain to a computer, BCI systems are typically divided into three categories: *active, reactive*, and *passive*. For a somewhat more precise division based on the mode of operation, BCIs are also often categorized into different *paradigms*, implicitly specifying if it is used in an active, reactive, or passive system.

#### 1.2.1. Active BCIs

With an active BCI the subject is intentionally trying to modulate mental states, for example by actively thinking *left, stop*, and *forward*. The goal of intentionally encoding such mental states is to generate signals that can be separated by the BCI system, and thus, subsequently can be used as instructions or inputs to some application.

A classic example is the *motor imagery* (MI) paradigm where the subject is imagining the movement of different parts of the body, without actually moving them (Abiri et al., 2019).

#### 1.2.2. Reactive BCIs

Another way of encoding information is to present different stimuli to a subject and then use the reactions to infer possible intentions of the subject, a *reactive* BCI. Typically, the subject pays selective attention to some stimulus (or category of stimuli) corresponding to some information desired to convey. The BCI then tries to discriminate the brain signals corresponding to the category of target stimuli.

A commonly used paradigm is the *oddball* paradigm, where stimuli of different categories are sequentially presented to the subject (Abiri et al., 2019). Here, one of the occasionally displayed stimuli categories is the target category which in some way, at least from the subject's perspective, is different from the other categories. As a result, different *event-related potentials* (ERPs) patterns are elicited depending on whether the subject is focusing on, or recognizing a certain category or not. The difference in brain signal patterns, time-locked to the stimuli onset, makes it possible for a computer algorithm to distinguish and classify the target category from the other, non-target categories. A common application is the P300-speller where different letters are flashed sequentially, and the subject is waiting for a certain letter to be flashed. Being able to decode a letter of interest and then repeatedly apply the process to new letters makes it possible for the subject to spell out words (Farwell and Donchin, 1988).

Another reactive BCI paradigm is the so-called *steady-state evoked potentials* (SSEP). Here, several stimuli (often visual) are oscillating at different frequencies, for example a number of flickering LED lights. The subject is asked to focus on one of the stimuli, corresponding to some information or command to be conveyed. If the subject gazes at the flickering stimuli, brainwaves are elicited with the corresponding frequency and its harmonics, as described by Muller-Putz and Pfurtscheller (2008).

#### 1.2.3. Passive BCIs

The final category is *passive* BCIs. Here, brain activity is monitored passively, i.e., without the subject's active intention of communicating with the BCI. Typical use cases would be to monitor the subject's attention, level of focus, cognitive stress, tiredness, or workload.

### 1.3. Next generation BCIs

Functional neuroimaging techniques are widely used for medical purposes to assess brain health and for disease diagnostics. These techniques also serve as a fundamental component in the field of cognitive neuroscience by offering valuable insights into the underlying mechanisms through which the brain enables cognitive functions. Such research comprises experimental paradigms designed to isolate the neural mechanisms supporting a particular cognitive function. In such experiments, participants are typically presented with multiple stimuli (e.g., faces and objects) and instructed to perform a cognitive task (e.g., memorize). Statistical analysis is conducted both at the participant level, contrasting neural data from different trial types, and at the group level testing hypotheses about population data. When analyzing the recorded brain signals to draw conclusions after the experiments, it is usually enough to know the onset-time and duration, and which stimulus was presented. For these purposes, during the experiment itself, it is sufficient to present a pre-determined sequence of stimuli. By pre-determined in this context we mean that the stimuli-environment is static and does not get adjusted during the experiment based on the subject's actions or decoded state of the brain. Such a static stimuli-environment means that stimuli-sequences and instructions could be chosen before the start of the experiment, either manually, randomized, or algorithmically arranged.

The purpose of a BCI is a bit different, seeking to convey information in order to, in some way, impact the state of the world. Similar to neuroscientific studies on brain functionality, when considering a BCI, a fundamental task is to discriminate between different brain states. However, in the case of BCI, not only by evaluation of statistical significance, but also while being as fast as possible. The desire for fast near real-time analysis originates from the idea that the results of the signal-decoding are used to interact with the surrounding world here and now, not hours or months later when all data has been recorded, cleaned, and carefully analyzed by offline methods. For this reason, the somewhat different nature of a BCI operating in near real-time, compared to traditional neuroscientific experiments, will put different requirements on the system in use. This will also influence the paradigm used to encode discriminable brain signals, as well as the methods and algorithms used for signal processing and analysis of the recorded neuroimaging data.

Since fast discriminability between mental states is desired when considering a BCI system, the paradigms used are typically more crude than regular neuroscience experiments. The regular paradigms used for BCI (briefly described in Section 1.2), for example, ERP oddball, motor imagery, and SSEP, are all designed to create maximum separability between different experimental conditions. For each paradigm, the neural mechanisms used and detected are typically the same for any application, not taking into account if the used encoding is a natural way of transferring information or not. A less crude way would be to better align the way information is conveyed with human intuition of the task at hand. A simple example would be playing a game where you can jump and go forward. For a human, it is probably more natural to imagine walking and jumping rather than imagining moving the right and left arm respectively. Of course, tailoring the decoding algorithms to such encodings would probably put completely different computationally and algorithmically requirements on the system, compared to the BCIs of today.

Not only the paradigms are different when comparing BCI system with more traditional neuroscientific studies. While neuroscientific experiments are mostly focused on understanding how the human brain works on a population level, with a BCI we are interested in enabling each individual subject to convey information as fast as possible. Thus, it also makes sense to, if possible, individualize the analysis as much as possible. This aspect is reflected not only in the use of machine learning for the classification of data, but also by using data-dependent methods for individualized feature extraction such as *common spatial patterns* (CSP; Koles, 1991), and xDAWN (Rivet et al., 2009). Using data-driven methods enables the use of transfer learning, where knowledge or data from analyzing one problem is applied when trying to solve another, related problem. In the case of a BCI, this would typically be to use data from other subjects, sessions, and experimental paradigms. Because of the well-known inter-subject and inter-session variability in EEG data, is is highly desirable to transform and transfer data, to make the data generalize better across different conditions.

The utilization and empirical exploration of transfer learning provides an opportunity to leverage larger and more diverse datasets, consequently facilitating the usage of advanced data-driven models. Additionally, as more data in the current session becomes available it is possible to gradually improve models or switch strategy to optimize the performance of the BCI system during use. Bigger datasets and more advanced and dynamical models may require more computational resources, which can be handled by offloading heavy computations to cloud resources.

**Figure 2:**
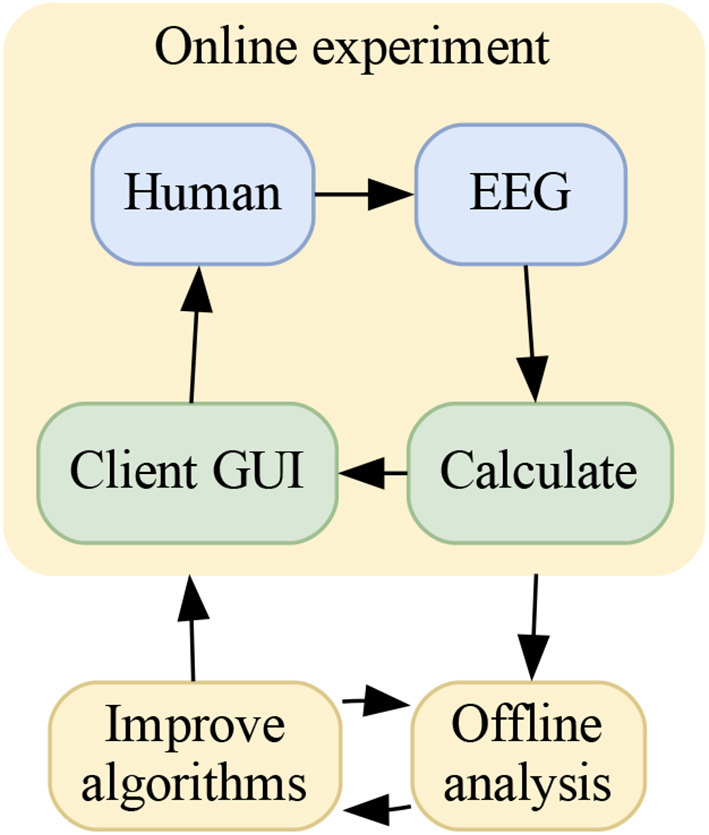
A dual loop present in many online BCIs, showing how to improve the human-in-the-loop BCI performance. The inner loop is the online experiment where the human subject and the BCI interact. This is where both of them learn how to collaborate and work together. The outer loop is where this interaction can be analyzed in detail to improve the BCI part of the inner loop.

Another important aspect is closing the loop with the human using the BCI. It is natural to consider this aspect when developing BCI systems, as the near real-time analysis might be used to dynamically alter the stimuli-environment. In contrast to static stimuli mentioned above, dynamic stimuli would mean that the environment is changing based on analysis of the brain states in near real-time. With the goal of using a BCI to convey information in order to change the state of the world, it makes sense to also develop algorithms in a human-in-the-loop setting where the stimuli-environment is influenced by the information decoded by the BCI. This aspect might be especially relevant for an active BCI, since modulating the mental states is a somewhat continuous task, to a high degree self-inflicted. It is shown that in such scenarios, the subject would experiment with the way mental states are encoded, either if the output results or commands are not satisfactory, or just slowly drift in strategy over time. Dynamic stimuli might also be beneficial when considering reactive BCIs, where specific stimuli can be presented to the subject in order to optimize some quantity of interest, as described by Tufvesson et al. (2023). Finally, considering that additional insights gained from offline analysis can be used to improve the online analysis and experimental setup, we see an emerging dual loop for improving BCI performance. An illustration is given in Figure 2.

When comparing aspects of neuroscientific studies trying to understand the mechanics of the brain, and the development of BCIs, there are both differences and possible synergies. Using knowledge and insights from neuroscience could be essential for developing more advanced BCIs, when combining data-driven methods such as machine learning and individualized feature representation with more advanced models on how information is processed inside the brain. In the other direction, more advanced algorithms and signal representations developed for near real-time analysis in BCI could help neuroscientists to perform more advanced analysis and make use of bigger data sets, faster computational resources, and dynamic experiments.

In summary, the development of advanced BCI systems requires consideration of various aspects, including the neural mechanisms of the brain, computational resources for data-driven algorithms, and human-in-the-loop capabilities to cope with dynamic stimuli-environments. The optimal combination of these components for development of an efficient BCI system across different paradigms remains unclear. Hence, in order to facilitate the evaluation and testing of advanced approaches to BCI systems, it is crucial to test various options. This process is made easier when the software and hardware are both user-friendly and highly customizable. The BCI-HIL framework is in active development and is written in modern high-level languages using freely available tools.

### 1.4. Outline

For readers interested not only in design principles and a system overview, but also in implementation details and programming, we recommend cloning the provided code repository containing all code used in the paper. Inspecting the actual code in parallel with reading the paper will lower the level of abstraction while also providing more insights and inspiration. For instructions on how to set up and run the examples, we refer to the README.md-file located in the root folder of the BCI-HIL repository.

In Chapter 2, Materials and equipment, we describe hardware and software tools relevant when designing or considering using a BCI framework, for example EEG-caps, communication protocols, and software for stimuli presentation and signal processing. In Chapter 3, Methods, we first describe desirable properties of a BCI framework and then show how open-source software tools can be used to design components of a human-in-the-loop BCI with these objectives in mind. This is followed by some practical aspects to consider when using a BCI. In Chapter 4, Results, we present and provide two BCI applications designed using the BCI-HIL framework. The paper is concluded with a discussion in Chapter 5.

## 2. Materials and equipment

Some major system components are found in almost every BCI. First of all, equipment for signal acquisition of functional neural activity is required. Additionally, in order to receive, record, and/or analyze the measured signals, a computer with relevant software is needed. In many cases one is also interested in hardware and software used for providing a controlled stimuli-environment. In this section, different hardware and software-tools relevant when designing a BCI framework are presented. Extra focus is given to components that will be used as sub-components of BCI-HIL, presented in Section 3.2.

### 2.1. Measure functional neural activity

There are many possible ways of performing functional imaging of neural activity in the brain. Some technologies directly measure activity in the electric domain while others use hemodynamic-based measures (indirect measures of neural activity based on properties of the blood in the brain). The most prominent technologies used in the electric domain are *electroencephalography* (EEG; Berger, 1929) and *magnetoencephalography* (MEG; Cohen, 1968), measuring electric field potentials and magnetic fields, respectively. Correspondingly, for hemodynamic-based measures two of the most common technologies used are *functional magnetic resonance imaging* (fMRI; Belliveau et al., 1991) relying on *blood-oxygen-level-dependent* (BOLD) contrast (Ogawa et al., 1990), in turn dependent on paramagnetic properties of hemoglobin, and *functional near-infrared spectroscopy* (fNIRS; Jöbsis, 1977) which measure changes in hemoglobin concentrations.

There are also many other methods for functional neuroimaging, such as *positron emission tomography* (PET), *intracortical neuron recordings* (INR), and *electrocorticography* (ECoG; Leuthardt et al., 2004), where the last two are of invasive nature.

In general, there are fundamental trade-offs that are made when choosing any of the above-mentioned technologies used for functional neuroimaging. Examples of these trade-offs are temporal and spatial resolution, price, portability, ethics, personal health risks, and ease of use. In this paper, we focus on EEG which excels in terms of temporal resolution, price, and portability, as well as low risk from a health perspective. However, compared to many of the other technologies, EEG lacks substantially in terms of spatial resolution (Nam et al., 2018).

### 2.2. Landscape of EEG processing tools

There are many available tools and frameworks intended for various types of EEG-recordings, paradigms, experiments, signal processing, and *post-hoc* analysis. Listed below are some tools commonly used for EEG-analysis, and to some extent for other functional neuroimaging technologies. Firstly, tools specifically designed for real-time analysis are presented, including frameworks that have been widely utilized over an extended period and some more recent alternatives. Then, some tools mainly targeted for offline analysis are presented, and finally, a couple of frameworks used for stimuli-presentation are covered.

Regarding open-source licenses, the MIT and BSD licenses are the least restrictive, with no implication on patents, and modifications to the original source code can be made without requiring derived works to be open-sourced as well. The MIT and BSD licenses exists in many versions.^1^ The GPL license,^2^ which is also common in open-source software, puts some additional requirements regarding patents and forces derived works to publish any updated source code as open-source as well.

#### 2.2.1. Real-time online BCI research frameworks

##### 2.2.1.1. BCILAB

BCILAB^3^ is an open-source MATLAB-based toolbox as described by Kothe and Makeig (2013) with a GPL license. BCILAB is designed as an EEGLAB^4^ plugin used for design, prototyping, testing, experimentation with, and evaluation of brain-computer interfaces. The toolbox was maintained from 2006 to 2017 and is no longer in active development.

##### 2.2.1.2. FieldTrip

FieldTrip^5^ is an open-source MATLAB software package for analysis of MEG, EEG, and electrophysiological data as described by Oostenveld et al. (2011). The toolbox has been developed since 2003 and is released under a GPL license.

##### 2.2.1.3. BCI2000

BCI2000^6^ is a general-purpose software system for brain computer interface research as described by Schalk et al. (2004), released under a GPL license. BCI2000 includes software tools that can acquire and process data, present stimuli and feedback, and manage interaction with outside devices such as robotic arms. BCI2000 is written in C++ with interfaces to MATLAB and Python in Microsoft Windows, with limited functionality running on other operating systems.

##### 2.2.1.4. OpenViBE

OpenViBE^7^ is a software platform as described by Renard et al. (2010) with an AGPL-3 license, that enables to design, test, and use of brain-computer interfaces. OpenViBE can also be used as a generic real-time EEG acquisition, processing, and visualization system. OpenViBE was actively developed between 2006 and 2018. It supports Microsoft Windows, Ubuntu, and Fedora.

##### 2.2.1.5. Falcon

Falcon^8^ is a highly flexible open-source software for closed-loop real-time neuroscience as described by Ciliberti and Kloosterman (2017). Falcon is written in C++ and is released under a GPLv3 license.

##### 2.2.1.6. Gumpy

Gumpy^9^ is an open-source toolbox for development of BCI systems. It is written in Python and is mainly based on a collection of already proven Python libraries such as NumPy, SciPy, and scikit-learn as described by Tayeb et al. (2018). Gumpy is released under the MIT license.

##### 2.2.1.7. Timeflux

Timeflux^10^ is an open-source framework for data collection and real-time processing of generic time series data. However, it is developed with BCIs and other bio-signal applications in mind. Timeflux is released under the MIT license and is written in Python, and can be used across platforms. The real-time processing capabilities in BCI-HIL presented in this paper are based on Timeflux. Thus, a more detailed overview of the framework is given in Section 2.4.4 below.

#### 2.2.2. Non-realtime offline BCI research frameworks

##### 2.2.2.1. EEGLAB

EEGLAB^11^ as described by Delorme and Makeig (2004) is an open-source MATLAB toolbox for analysis of averaged and single-trial EEG data. It is released under a GPL license.

##### 2.2.2.2. MNE-Python

MNE-Python^12^ is an open-source Python package for exploring, visualizing, and analyzing human neurophysiological data: MEG, EEG, sEEG, ECoG, NIRS, and more, as described by Gramfort et al. (2013). MNE is released under the BSD license.

#### 2.2.3. Stimuli toolboxes

##### 2.2.3.1. Pyff

Pyff^13^ is the Pythonic feedback framework released under GPLv2 license. Pyff uses the network protocol UDP to communicate with other modules in the BCI system, and XML is used to wrap arbitrary data in a format Pyff can handle, as described by Venthur et al. (2010).

##### 2.2.3.2. Psychopy

Psychopy^14^ is an open-source Python package with a GPLv2 license to build experiments using a GUI or a programming API.

### 2.3. Hardware

#### 2.3.1. EEG hardware

There is a plethora of hardware devices available for non-invasive EEG signal acquisition, ranging from open-source low-cost (Teversham et al., 2022) to high-end, wireless, and closed source. What device to choose depends on which type of research environment you target. Any hardware based on the Lab Streaming Layer (LSL), described in Section 2.4.2 is, compatible with BCI-HIL. We have implemented and tested the BCI-HIL research framework using three different EEG hardware devices: MBT Smarting, Muse S, and Neurosity The Crown.

##### 2.3.1.1. MBT Smarting

The MBT Smarting^15^ as introduced by Debener et al. (2012) is a wet electrode wireless EEG system. It uses a Bluetooth transceiver to send EEG signals to an Android smartphone that can re-transmit the EEG stream, the head accelerometer, and smartphone accelerometer data in LSL outlets. It has 24 EEG electrodes evenly distributed across the skull, and a 3 degrees-of-freedom accelerometer. The sampling rate is either 250 or 500 Hz, and the companion Android control app can display measured electrode impedances during cap appliance.

##### 2.3.1.2. Muse S

The Muse S^16^ headband is a low-cost Bluetooth wireless EEG device. Following the international 10–20 system, Muse S features four channels: frontal AF7 and AF8, temporal TP9 and TP10, as well as FPz used as reference. The electrodes are made of conductive ink on flexible fabric adhesive, and the data sample rate is 256 Hz. Additional sensors on the Muse S are accelerometer, gyroscope and photoplethysmography (PPG) heart rate sensor using LEDs. The device introduces an unwanted time delay in the range of 20–40 ms with 5 ms jitter, and a 0.01-0.05% loss of EEG samples, as described by przegalinska et al. (2018). Accessing data from Muse S over LSL can be done for example by using the muse-lsl Python package (Barachant et al., 2019).

##### 2.3.1.3. Neurosity The Crown

The Crown^17^ is a wireless EEG system using dry electrodes and wifi. It provides LSL signals directly from the hardware for the eight EEG channels, primarily located on the top of the head over the motor cortex, sampling at 256 Hz. Following the 10–20 system, the electrodes are placed at Cp3, C3, F5, PO3, PO4, F6, C4, and Cp4, with reference electrodes at T7 and T8.

### 2.4. Software

#### 2.4.1. Programming languages

A number of programming languages have historically been used for BCIs. C++ and MATLAB^18^ were some of the first, while more modern alternatives have emerged, replacing for instance the commercial MATLAB language with Python,^19^ much due to the fact that Python is free to use and have a large open-source community developing frameworks like MNE-Python. On the far horizon, the Julia^20^ programming language is rising, addressing some of the drawbacks with Python like slow computations and dependency on optimized C-code. However, Python and Julia are mainly scientific compute languages, and they are not the ideal candidates when it comes to visualization and stimuli presentation.

Today, JavaScript^21^ is the most widely used programming language^22^ for open-source software, and is used both for front-end and back-end web programming. Graphical user interfaces (GUIs) using HTML^23^ and CSS^24^, and the companion language JavaScript are easy to set up and require no compiling or building, which gives quick visual feedback. Using these programming languages there are many visualization libraries helping you to produce interactive graphics, both for 2D and 3D, to use Virtual Reality headsets, and replaying videos and audio.

#### 2.4.2. Inter-process communication

When building a modular software system, inter-process communication between different parts is needed for the subsystems to cooperate. Various methods have been used throughout computing history like *signals, message queues, sockets, named pipes*, and *shared memory*, to name a few. For a research framework, compatibility is highly desirable, both between operating systems but also between programming languages. Also, the possibility to run the subsystems on separate machines across a wired or wireless network needs to be considered.

##### 2.4.2.1. Lab streaming layer

The Lab streaming layer (LSL) is a system for real-time measurements and time-synchronization of time series data between various computers and input devices. LSL handles data-streams both with uniform sample rate such as EEG data, and non-uniform sampling rates such as event streams from a stimuli device, mouse-clicks, and other types of inputs. Communication over the LSL can be setup in a number of programming languages (C/C++, Matlab, Python, Java, etc.) with just a few lines of codes. Over the last decade LSL has been used extensively for EEG signal acquisition and online processing. As a result, LSL supports many EEG toolboxes and EEG caps, as well other input devices such as video game controllers and eye-trackers.^25^

Under the hood LSL is using network protocols such as UDP and TCP, and communication is facilitated using a core library called *liblsl* as described by Stenner et al. (2022), implemented in the C++ programming language. Besides C++, interfaces for programming languages such as Matlab, Python, C, Java, and Julia are also available, which makes it easy to use LSL in most scenarios.

In order to make data available to other devices and computers on the local network, a producer of data like EEG caps and stimuli programs create an *LSL-outlet*. An LSL-outlet contains metadata relevant for the particular source of data, and provides functions to push data out on the network. The combination of data and corresponding metadata is referred to as an *LSL-stream*. With the data stream available, other applications on the network can find a particular stream by looking for some specific field, information, or attribute in the metadata. When a stream with the desired attribute is found, an *LSL-inlet* is defined. The LSL-inlet is then used to acquire data that is made available on the network through the corresponding LSL-outlet.^26^ The LSL software also comes with an LSL-recorder written in Python that can be used to save data from all LSL-streams on the network during a specific session. This is useful to save the full sequence of events and data generated during a session. The data is saved in the *extensible data format* (XDF)^27^ file format.

With a wide support of programming languages and relevant devices, simplicity to use, community adaptation as well as being open-source, LSL is a natural choice for some parts of the inter-process communication in a BCI system.

##### 2.4.2.2. Websockets

Unfortunately, web technologies running inside a browser are not allowed to open raw sockets as those used by LSL. An LSL implementation could be implemented for server-side JavaScript based coding using node.js^28^ or similar back-end tools. However, the current security model for browser-based JavaScript does not permit the low-level network handling that is a vital part of LSL, as described above.

Rather than using HTTP polling, websockets is a full-duplex communication link permitting transfers to be initiated both from the client and the server once the websocket is up and running. This is used as a low-latency communication link to handle a real-time BCI for visualizations, stimuli presentation, and the subject's input and output. Websockets use TCP networking and work across operating systems, separate computers, and between processes run on the same computer.

#### 2.4.3. Stimuli software

For any experimental setup for human-in-the-loop BCI research one needs ways of presenting stimuli to the subject. Today's computers are pretty good at presenting images, videos, playing audio, and doing 3D graphics. For such stimuli there are ready made stimulus software tools and Python modules that can be used. For external stimuli like lights, USB-connected embedded electronics can be used. Using taste, smell, or haptic feedback is not so common.

Modern computer monitors typically have a fixed update frequency, typically 60 Hz, or higher when considering displays intended for gaming. The latency from a display software update to the actual update of the graphics on the screen will have a time jitter with uniform random distribution between 0 and 16.7 ms, on top of the fixed unknown graphics pipeline latency. One remedy to the jitter related to display refresh rate is to make sure that the lines of code in the software that updates the display are synchronized to the updates of the display. One such mechanism is the Window.requestAnimationFrame() that is part of the web APIs found in common browsers like Google Chrome, Mozilla Firefox, and Apple Safari, and access it using JavaScript. This event acts like an interrupt that will trigger every time the monitor updates, effectively synchronizing the stimulus presentation with the display. However, there is still an unknown latency between this software interrupt and the actual display update. A websocket callback can tell other parts of the system when the update happened. The inter-process communication will have smaller jitter compared to the display refresh jitter. Note that using advanced display modes, like turning off double buffering from the GPU, will not change the amount of jitter in the display update, it will simply lower the latency without affecting the jitter uncertainty. Actually, without double buffering the possibility of reducing the jitter using a display refresh rate interrupt is lost.

##### 2.4.3.1. Using modern web technology for stimuli presentation

Naturally, there are ways of playing audio and presenting images and videos to a subject in almost any programming language. However, modern web technology is cross-platform and surprisingly easy to handle for almost any kind of stimuli like audio, images, video, and virtual reality. Another benefit is the lack of compile time, providing instant feedback by a simple reload of the browser page. There is lots of help to be found, with numerous examples and guides on the internet. Virtual Reality stimulus can be implemented using ThreeJS and WebGL, supporting a wireless Meta Quest 1 and 2 wireless VR headset, as well as tethered VR headsets. These kind of web applications can easily be run on MacOS, Microsoft Windows, and Linux by installing the Google Chrome web browser.

#### 2.4.4. Online processing with Timeflux

Timeflux (Clisson et al., 2019)^29^ is an open-source framework for data collection and real-time processing of generic time series data, developed with bio-signal applications and BCIs in mind. The framework is written in the Python programming language. Timeflux is designed for being easy to use, having a lightweight core functionality, being modular in the sense that sub-components are replaceable, making it easy to reuse or incorporate existing code, and being easily extendable by adding custom or modified modules.

In this section, some fundamental concepts of Timeflux will be introduced and explained. For a more extensive presentation and documentation, see the original paper and the online documentation (Clisson et al., 2019).

##### 2.4.4.1. Timeflux basic concepts

Applications in Timeflux consist of one or multiple *graphs*, constructed from *nodes* and directed *edges*. Nodes are used to process data while edges define how and in which direction data flows between the nodes within a graph. All processing steps in one graph are executed at the same frequency, the *rate* of the graph. Different graphs can be executed at different rates and communication between graphs is facilitated by a publisher/subscriber system.

In Timeflux, the structure of the nodes and directed edges have to be defined in a way such that the resulting graph is a *directed acyclic graph*, meaning that no cycles can be formed. Thus, following the directed edges, it is impossible to get to a node of the graph that has already been traversed. The directed acyclic structure guarantees that the processing steps of each node can be executed sequentially, where certain nodes have to be executed before others, as the output of some nodes might be the input(s) to other nodes. The sequential execution also implies that the full sequence of processing steps in a graph can be executed at fixed frequency (rate) without ambiguities in order of execution.

A Timeflux node is a regular Python class with the addition of some inherited extra functionality from the Timeflux *Node* superclass, such as receiving data from and sending data to other Timeflux nodes within the graph. Receiving and sending data is done with *input ports* and *output ports*, which are inherited from the Timeflux Node class. Every node also has a function called update(). Every time a graph is executed, the update() function for each node of the graph is called once.

Communication between different graphs is done asynchronously using a publisher/subscriber system facilitated by a few Timeflux nodes designed for this purpose. These special nodes are the *Pub* and *Sub*, and *Broker* nodes. The Broker node acts as a mediator handling the passing of data and is always placed in a separate graph. The Pub and Sub nodes are incorporated in graphs as regular nodes, providing an interface to the Pub and Sub nodes of other graphs. Under the hood, these inter-graph communication nodes are using the ZeroMQ-protocol.^30^

##### 2.4.4.2. Building Timeflux applications from existing nodes

For a Timeflux graph the structure of nodes, edges, and execution frequency are specified in a *yaml* configuration file. In the yaml-file, an instance of a node is specified by a number of fields. Typically, these fields are: a unique identifier, the name of the Python class implementing the node, and possibly parameters passed to the constructor used to specify non-default behaviors of the node. Similarly, an edge is defined by two fields, *source* and *target*. Here, the source specifies the identifier (and output port) from a node sending data, and the target specifies the identifier (and input port) of a node receiving the corresponding data.

##### 2.4.4.3. Notable Timeflux nodes

The Timeflux package comes with a number of nodes and functions providing the essential building blocks for running nodes and building useful applications. With the aim of having a lightweight core in mind, functionality other than the most essential, such as various digital signal processing (DSP) nodes in Timeflux-DSP and a simple user interface (UI) node in Timeflux-UI, come as separate packages. Some Timeflux nodes, essential for building EEG-processing applications, are mentioned below:

- The *Sub* and *Pub* nodes are used to facilitate inter-graph communication by subscribing and publishing to so-called topics.
- The *Send* and *Receive* nodes (from the LSL module) are used to send and receive data to/from LSL-streams on the network.
- The *Epoch* node buffers and collects EEG data and then time-locks it to stimuli event markers, which indicate that a stimuli was presented to the subject. If the event marker also contains label information, this data is concatenated with the epochs such that labeled data used for machine learning can be easily constructed. The *Window* node has a similar purpose as the Epoch node. The difference here is that epochs are now cut with fixed time intervals, possibly overlapping, non-time-locked in relation to external events or stimuli.

##### 2.4.4.4. Building custom Timeflux nodes

Custom Timeflux nodes can easily be developed and implemented. As mentioned above, a node is a regular Python class inheriting a few properties and requirements from the Node superclass. Thus, the implementation of a custom node is very similar to implementing a regular Python class. The constructor arguments specify parameters that can or need to be passed when creating an instance of the node/class. Non-default parameter values are passed from the yaml-file, and the code inside the constructor is run once upon initialization. Then, the code in the update() function is run once every time sequence of steps in the corresponding graph is executed. For near real-time processing of data, the update() would typically consist of the following steps: First, check that some conditions of interest are fulfilled, for example if all input data of interest is available on the input port(s). If so, unpack the data and perform any desired operations on the data. Finally, send results to an output port, making it available to the next node in the graph.

## 3. Methods

In this section, we start by discussing desirable properties of a BCI framework. We then describe how some of the existing equipment, communication protocols, and frameworks presented in Chapter 2 could be used to design components of a BCI framework which we will later refer to as the BCI-HIL research framework. Chapter 4 will describe how these components can be combined to more specific BCI applications. Finally, practical aspects and limitations of latency calibration and real-time filtering are presented.

### 3.1. Desirable properties of a BCI research framework

There are many desirable properties of software artifacts such as functionality, ease-of-use, and customizability, and these apply to BCI research frameworks as well. There will always be trade-offs between different aspects of these properties as some are, or might be, in direct or indirect conflict with each other. What properties are most important will clearly depend on the intended use, and who is setting up and running the system.

#### 3.1.1. Functionality

One important property to look for is the current functionality of the framework. If features required for the intended use are not available, there are two options. Either move on to another set of tools or try to get the missing features implemented into the framework in some way. If this is possible will depend both on other properties of the framework such as customizability and community support, as well as the available resources and programming skills at hand. If the user of the framework has intentions to develop new custom functionality it is still important to evaluate the fundamental properties of the framework such that the desired extensions can be implemented without a complete redesign of the framework.

#### 3.1.2. Ease-of-use

What makes something easy to use is not the same for people of different backgrounds. It also depends on what aspects of the system that should be easy to use. Should it be easy to get started, to build standard BCI paradigms, or to implement new algorithms? In general, the framework should: be quick and easy to install, have intuitive setup and usage, have a GUI, enable use of standard equipment and interfaces, be compatible with already existing hardware and software, have possibility to add not-yet-released hardware, etc. Additionally, a research framework should preferably not require expensive commercial licensing of closed-source software.

#### 3.1.3. Modularity

Using a modular design with standard interfaces between components is important if the intended usage of the framework is changed, if better alternatives to some parts of the system become available, or if some new equipment needs to be added. Should any part of the framework require modification or supplementation, it would be advantageous for the specific component in question to be able to be altered independently. Another important aspect of a modular design is that system components that are not used can be removed, allowing for an application with as low resource requirements as possible when it comes to computations, memory requirements, and hardware cost.

#### 3.1.4. Compatibility

Another desirable property for the system is to be compatible with as much relevant software and hardware equipment as possible.

#### 3.1.5. Customizability

Having a system that can be customized is, in some contexts, also highly desirable. Some properties that make a framework customizable are modularity (see above), the code being open-source, and the ability to build and incorporate custom system components in a frictionless way.

### 3.2. BCI-HIL modules

With the overall ambition of providing and exemplifying tools for researching and developing the BCI systems of the future, we present a system design with the main objective of being a modular framework that is fully and easily customizable. We also aim for a design using only open-source components that can be run on any of the most common operating systems (Windows, MacOS, and Linux), and distributed on multiple computers if desired.

With these objectives in mind, visualizations and graphics are displayed directly in one or several web browsers. Real-time features are provided by the Python package Timeflux, while signal processing and machine learning functionalities can be either implemented from scratch or fully performed by (or combined with) standard packages from the Python community, such as SciPy (Virtanen et al., 2020) and scikit-learn (Pedregosa et al., 2011). A central module is keeping track of the dynamics of the stimuli environment and high-level logic for signal processing and machine learning. Communication is enabled via standardized technologies, such as websockets connecting modules, and LSL facilitating the transfer of EEG data and stimuli streams between the modules and associated hardware.

**Figure 3:**
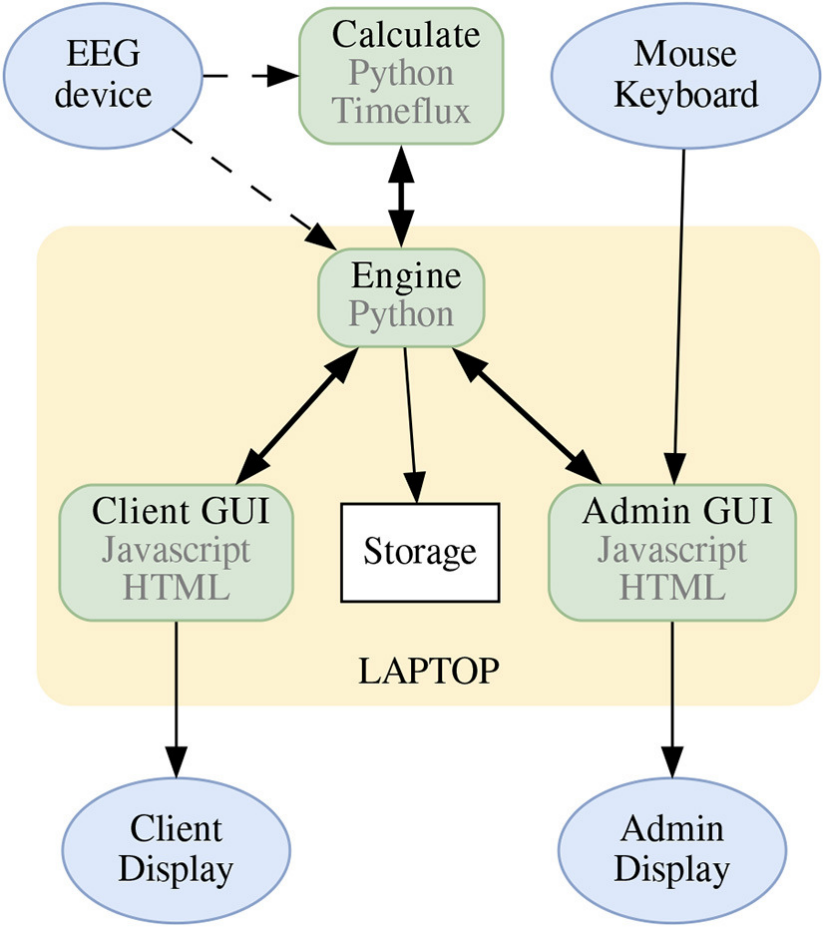
The hardware block schematics of a BCI-HIL research setup. The *Engine* is the central software knowing the state of the experiment, gathering and sending commands to/from the other submodules. The *Client GUI* and *Admin GUI* take care of displaying stimuli and information, and the *Calculate* program could handle online machine learning, inference, classification, and transfer learning. The dotted lines indicate communication using LSL, the bold bidirectional arrows represent communication using websockets, and the remaining arrows show communication using USB/HDMI. Note that the mentioned software modules can be run using separate computers if needed. The blue elliptical nodes are input and output devices to the BCI-HIL research framework.

The BCI-HIL framework has a modular build, with well-defined inputs and outputs between the modules. This enables us to replace parts, combine different programming languages and get an advantage by using the most fit tools for their purpose. The modules are the *Engine*, the *Admin GUI*, the *Client GUI(s)*, and the *Calculate* program, as seen in Figure 3. They are described in more detail below.

#### 3.2.1. The Engine program

The Engine is the part of the BCI-HIL research framework that knows everything. It provides time synchronization in sub-millisecond precision to all other modules in the system. It keeps track of experiment state and relays information between the Admin GUI, the Client GUI, and the Calculate program. Additionally, the Engine creates relevant LSL marker events indicating stimuli onsets and other experimental conditions. For different BCI applications, some parts of this program have to be re-designed to enable the desired behavior. For example, BCI paradigms like P300, Motor Imagery, and SSVEP all require different experimental setups which need to be implemented in the Engine program. However, interacting with the other modules of BCI-HIL will look very similar between applications. Finally, the Engine program archives the incoming EEG data and LSL events on a local disk, to facilitate subsequent offline analysis.

#### 3.2.2. The Admin GUI

**Figure 4:**
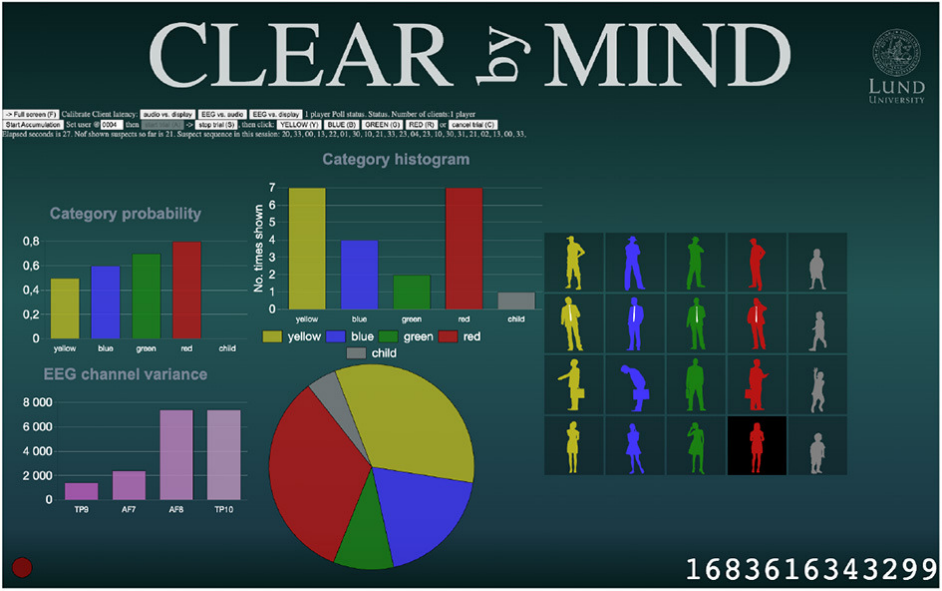
Screenshot of an Admin GUI. The Admin GUI controls the BCI experiment through clickable buttons and keyboard shortcuts while visualizing the current state and classifier output.

The Admin GUI is where any control command is given by the experiment administrator. This is done from a series of action buttons like *start trail, pause trail, cancel trail*, as well as text fields where information such as the subject-ID, session number, and other experiment specific data can be input. The Admin GUI is also where online experiment feedback is shown. This could be anything from raw EEG-signals to graphs like classification probabilities, stimuli histograms, visualizations using dimension reduction, or scatter plots. The information presented here is meant to supervise the inner workings of the algorithms in order to help the researcher understand and improve the experiment setup. Figure 4 shows a screenshot of the admin GUI in the Clear By Mind application (further details in Section 4.2). Additionally, the internal timestamp of BCI-HIL is shown on the Admin GUI display. If the experiment is video-recorded, this timestamp can be used to match recorded data from the LSL-streams with real-world experimental conditions, allowing for backtracking of potential issues or locating events of interest.

#### 3.2.3. The Client GUI(s)

**Figure 5:**
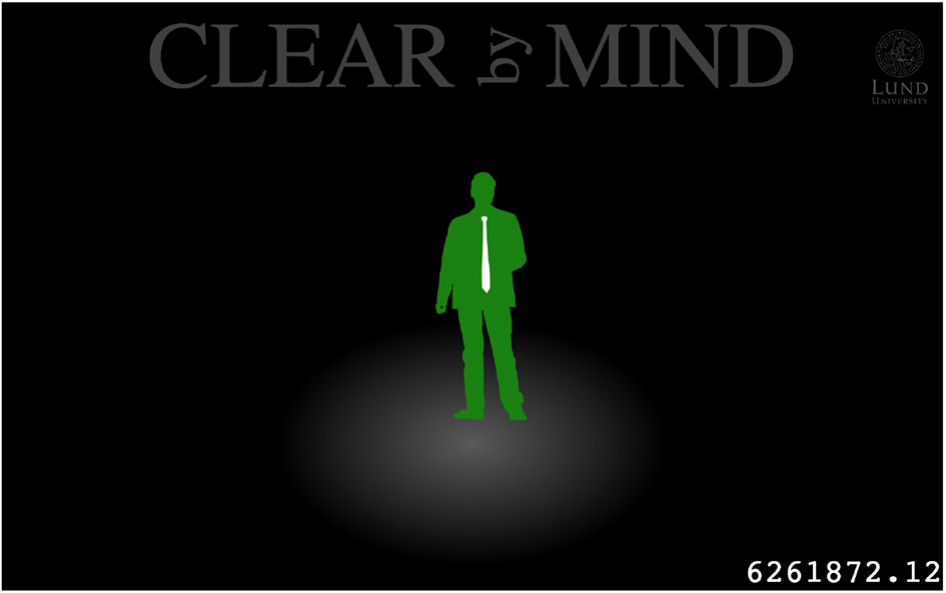
Screenshot of a Client GUI, in this case from the BCI-HIL Clear By Mind example application. Here, the subject is asked to focus on some category of visual stimuli, which changes a few times per second during a session (see Section 4.2 for details).

The Client GUI is where the subject is focusing and where stimuli are presented during a trial. Connected to a monitor the module can show visual stimuli as typically done in many BCI paradigms. Similarly, with speakers connected, the Client GUI can also produce audio stimuli for the experiment if desired. Figure 5 shows a screenshot of the client GUI in the Clear By Mind application (further details in Section 4.2). This module is synchronized to the display output, to keep the jitter of visual event markers low. Several Client GUIs can be run at the same time, either on the same or different computers connected the wifi network. This enables interactive sessions with multiple subjects, such as competitive games or collaborative tasks.

Similar to the Admin GUI, the Client GUI displays the BCI-HIL timestamp, enabling time synchronization of video recordings with timestamps of data recorded from the LSL-streams. However, to reduce the cognitive distraction of the subject, this timestamp only updates at events and does not run continuously.

#### 3.2.4. The Calculate program: Timeflux in BCI-HIL

In BCI-HIL the Timeflux application is constructed using four graphs: input/output, preprocessing, save, and machine learning graph. The first one is listening and sending data to relevant LSL-streams. In our case the LSL-streams of interest are the raw EEG data, stimuli markers, and messages with status and instructions from the Engine program.

The preprocessing graph is where the EEG time-series is filtered and cut into epochs of appropriate length. The epochs are either cut based on a stimuli onset time, or with a fixed time interval in a rolling window fashion, possibly overlapping. In the former case the epochs might also be paired with the corresponding labels from the stimuli markers. The structure of this graph would look similar for most EEG-related applications. Parameters that might be of interest to tailor based on application and paradigm in this graph are: type and cut-off-frequencies of band-pass filter, epoch length, and whether to use epochs time-locked to stimuli or not. Other nodes that could make sense to have in this graph are artifact removal and baselining. The save graph continuously archives epochs and corresponding labels (when applicable) to disk.

The machine learning graph performs various kinds of analysis on the preprocessed data. The structure and nodes used in this graph will depend heavily on the paradigm, application, and what aspects of the BCI that are of interest at the moment. For combined calibration and inference sessions it is natural to first collect and save labeled data. Then, when ready for a feedback session, use the collected data and possibly additional data from a cloud database, to train a machine learning pipeline of choice, and finally apply the trained model to new epochs.

##### 3.2.4.1. Custom BCI-HIL nodes

In order to facilitate signal processing and machine learning algorithms in line with design principles described above, a few Timeflux nodes that are not part of the original Timeflux package were implemented. These custom nodes would be a natural part of many BCI applications, and they are implemented to facilitate easy integration of transfer learning and custom functionality.

- **TrainingML:** Upon request, this node trains a scikit-learn pipeline using the collected data from the session (and possibly other data) and saves the trained model locally to disk.
- **InferenceML:** This node loads a trained scikit-learn pipeline from disk and runs inference on new EEG-epochs made available from the preprocessing graph.

Scikit-learn is one of the most widely used machine learning packages using the Python programming language. The package includes ready-to-use implementations of a large number of tools and algorithms for machine learning and signal processing. Scikit-learn uses a standardized syntax to specify how different models transform data and how they can be trained on data. This format is so common that the majority of third-party machine learning and signal processing algorithms, even those not included in the scikit-learn package, also implement the same structure. This makes a large number of algorithms from the whole Python community available, for example all decoding modules from MNE-Python. The standardized structure allows several modules to be piped together, acting as one module, using a scikit-learn pipeline, enabling fast prototyping. Enabling intuitive usage of scikit-learn compatible modules in BCI-HIL gives direct access to many off-the-shelf algorithms as well as an easy way to implement and integrate custom modules, in turn leading to easy experimentation, ease-of-use, modularity, and customizability.

With the BCI-HIL framework a researcher can add their own machine learning algorithms to the Timeflux nodes as per the requirements in their experiment, as long as it follows the scikit-learn syntax. BCI-HIL provides the structure for presenting the experiment, collecting and analysing the data but the user can adjust all parts of the framework to their needs. This is one of the strengths of the BCI-HIL framework, the customizability for the user to implement any BCI paradigm and corresponding machine learning algorithm they need.

#### 3.2.5. BCI-HIL advantages

Our research framework only depends on free-to-use open-source tools and languages. Other state-of-the-art BCI frameworks such as BCILAB, EEGLAB, and FieldTrip requires licensing MATLAB, and Octave being a somewhat MATLAB-compatible environment is unfortunately not mature enough to handle these extensive packages. Regarding programming languages, BCI-HIL is written in the Python and JavaScript languages, regarded as easier to learn and more portable than the C++ language used by the BCI2000 and Falcon frameworks. Also, BCILAB, OpenViBE, and Gumpy are no longer in active development.

### 3.3. Human-in-the-loop feedback

Traditionally, neurofeedback has been studied through the feedback of one-dimensional features, often based on the energy content within a specific frequency band. The goal is for the subject to consistently amplify or attenuate this feature. The purpose of many such studies has been to mitigate neurological disorders such as anxiety, insomnia, or epilepsy, or to improve desired states/behaviors such as cognitive performance or sleep quality. If such effects can be attributed to the modulation of certain neurological processes is still under debate, according to Marzbani et al. (2016).

Other studies have focused on providing neurofeedback with the aim of guiding the user to encode mental states useful for control of some kind of application, for example a simple computer game or moving a cursor on a computer screen, as done by Neuper and Pfurtscheller (2010). Additionally, it is well-established that brain activity and mental states can be decoded to varying degrees of accuracy, depending on the paradigm, equipment, and experimental setup utilized.

In an active BCI setting, the performance of the system is clearly dependent on both the BCI decoding algorithm as well as the encoding of mental states performed by the user, exemplified in a longitudinal study leading up to the Cybathlon BCI race (Perdikis et al., 2018). With the large inter-subject and inter-session variability seen in BCI, modern decoding approaches often take a data driven approach based on machine learning. Thus, the BCI is learning from data and can be tailored to a specific subject or session as more data becomes available. This leads us to the so called *co-adaptation* (Perdikis and Millan, 2020) and *two-learners problem* problem (Müller et al., 2017) where the machine and human both learns and adapt their strategies over time, hopefully converging to a system that can convey more information in a more robust and intuitive way.

Here it is important to give, not only the computer but also the human, relevant feedback such that the mental strategy can be adapted and/or learned. Thus, selecting what kind of feedback to display, and how, is of interest for optimal user-learning (Roc et al., 2021).

Additionally, for reactive BCIs with stimuli presented sequentially to the user, closing the loop with the human also has potential benefits. If the decoding results of stimuli presented early in the experiment indicates a certain result, this information could be leveraged to better decide which subsequent stimuli to display, as presented by Tufvesson et al. (2023).

In the case of a passive BCI, closing the loop with the user would be accomplished differently. Although the user is not actively attempting to communicate with the BCI, the results obtained from decoding can still be utilized to influence the environment in which the user is operating. Examples could be adjusting the difficulty of task based on decoding of cognitive load, or indicating when it is time to take a break due to tiredness.

### 3.4. Hardware latency calibration

When conducting neuroscientific experiments or using a BCI, it is often important to accurately synchronize stimuli onsets with the corresponding EEG-data. Different types of equipment for stimuli-presentation have different properties and imposes different types of delay. Also, within the same type of equipment there is a lot of variability. In order to mitigate the effects of intrinsic delays of the system components it is essential, for each setup, to perform a delay calibration. A thorough description of latencies in a BCI setup is presented by Wilson et al. (2010).

Another type of latency is the one introduced by signal processing steps, and an intrinsic problem when performing analysis on epoched data is that the signal processing cannot start before the data from the whole epoch time window is available. To get a faster response, an epoch has to be constructed from a shorter time window of data, which then can be processed with a lower latency.

#### 3.4.1. Calibrating audio to display latency

The combination of a chosen display device and an audio device will need to be calibrated. With human-in-the-loop calibration, this step includes adjusting an on-screen slider that will change the audio-to-display latency until the audio clicks are perceived to correspond to visual flashes on the display.

In order to obtain an approximate estimate of the intrinsic audio latencies present in your Client GUI setup, it is possible to conduct a measurement of the round-trip audio latency of the hardware and stimuli web application combined.^31^ Do note that this test, run in a web browser, measures your combined audio output and input latency.

#### 3.4.2. Calibrating display to EEG latency

The latency in a computer system from the CPU to visible changes on a display consists of many steps. The, much simplified, chain is from the CPU to graphics drivers, to the GPU and then to the display device. The latency in this part will change depending on settings like which render mode the OS uses (DirectX, OpenGL, Vulcan, Metal, etc.), as well as settings like double/triple buffering, vsync, and frame rate limiting.

Even though the Client GUI computer is good at knowing when the information is sent to the display system, there is still a part of the display latency that cannot be measured without external hardware.

The display used can be the internal display of a laptop, or an external display connected with any common interface like VGA, DVI, HDMI, Display Port or USB-C. Typical input lag values for HDMI input for an external computer monitor is between 9 and 117 ms, with a 29 ms median value, when tested using a Leo Bodnar HDMI input lag tester^32^ according to the Display Lag Database.^33^

For a TV the input lag is between 18 and 177 ms, and 9 ms to 31 ms in low latency game mode when measuring a range of hundreds of TV models.^34^ This kind of uncertainty needs to be accounted for, especially if we are to regard the higher time resolutions found in EEG signals. Also, note the difference between response time, input lag, and refresh rate. The response time for a TV or computer monitor is how fast one single pixel can flip from being light to being dark, while input lag is the time it takes for the monitor from first input signal to presenting the image on screen. The input lag is always larger than the response time. The refresh rate is how many times per second the display can redraw the image.

A calibration of a certain hardware setup needs only to be done once, then the found latency needs to be applied to all signals acquired with that setup. Numerous third-party stimulus trackers are available for this kind of latency measurements. As an alternative, we suggest using human-in-the-loop synchronization, where tapping the EEG headset to create artifacts in sync with visual flashes lets us measure the latency, at least to the accuracy of the human-in-the-loop's taps on the physical EEG hardware. Humans can tap to a beat with ~30 ms variance (Tierney and Kraus, 2013), and by measuring multiple taps, the variance can be averaged out.

#### 3.4.3. Calibrating audio to EEG latency

Playing sounds using a laptop includes a ring buffer of samples that is filled in an event-based interrupt driven function. Different operating systems and audio codecs will have different sizes of their audio buffers, and latency will depend on these factors. Using an external audio hardware digital-to-analog converter (DAC) will also change the latency, and using wireless headphones or an external amplifier will also add to the audio latency. Another factor to consider is the speed of sound, ~343 m/s in dry air at 20 °C, which introduces a latency of ~3 ms per meter from a loudspeaker to the subject's ears.

The setup used for getting the display to EEG latency is used similarly for audio to EEG calibration. Tapping the EEG headset to create artifacts in sync with audio clicks lets us measure the latency, at least to the accuracy of the human-in-the-loop's taps on the physical EEG hardware, as described in the previous section.

### 3.5. Hiding latency

The subject expects the Client GUI to update smoothly, with graphics being animated in sync with the Client GUI display many times per second, especially in a fast-paced BCI setting. The Engine program decides the state of the BCI system at a lower frequency, and any classification or machine learning might take even longer time and provide updates less often. One major challenge in such a system is to keep the subject immersed by hiding the slower parts of the system, keeping consistent graphics updates despite uncertainty in when results and classifications get updated.

Online multiplayer games have exactly this problem, where clients have inconsistent and varying latencies to the server. One approach used in multiplayer online gaming is to use predictive algorithms to extrapolate other players' movements and then correcting them every time the true server state arrives. Another strategy is to use techniques such as data compression and network optimization to reduce the amount of data that needs to be transmitted over the network. This can help to minimize the impact of network latency and reduce the amount of time that it takes for data to travel between the player's device and the game server logic. To minimize network congestion and latencies, use a local network to connect devices, dedicated to the computers running the experimental setup with as few as possible external devices present. Similar approaches could be used to keep the Client GUI updating smoothly.

A simple way to hide latency in a single-person BCI research setup is to smoothly animate changes in bar graphs and fade images in and out. Not everything should be smooth, though, since when using the P300 response as described by Chapman and Bragdon (1964), images shown to the subject should be shown instantly, to keep the onset event timing as distinct as possible.

### 3.6. Non-causal filters vs. causal filters

When doing offline analysis, we have access to all data in the EEG time-series. This helps us as non-causal filters with perfect frequency and phase responses can be designed. However, when using this type of filter for online processing, we have to wait for future signals to arrive, thus adding additional delay to the analysis. The other option is to employ causal filters, which inevitably entail a drawback or compromise that we need to take into account. A common way of making this trade-off is to use the impulse response of the desired non-causal filter, and time-shift it and multiply it using a windowing function, thereby truncating the length of the non-causal filter, turning it into a causal filter with an inherent delay. Good practices and what to avoid is discussed in the paper VanRullen (2011). The paper presents a warning against improper use of filtering, showcased with EEG signals shaped as step-functions with Gaussian noise and a filter function with excessive ringing. However, biological signals are rarely shaped as step functions and usually have lower noise levels than the signals in the paper. Nevertheless, the paper proves an important point. Filtering of event-related potential (ERP) onsets and distortion of data is commented by Rousselet (2012). Further insights on aspects of ERP filtering can be gained by reading about, and understanding, the basics of ERPs (Luck, 2014).

## 4. Results

In Sections 4.1 and 4.2 below, we provide examples and describe how the different system components presented in Section 3.2 can be combined and used to design two different BCI applications. The first application is a scaled-down motor imagery experiment, containing one calibration session and one feedback session. The second application is an unsupervised visual P300 task, where the goal is to distinguish images in a target category from images in a number of non-target categories. These detailed demos using the BCI-HIL framework with source code and instructions for running the applications can be found in the repository complementing this paper.^35^

### 4.1. Motor imagery BCI application

In this section, we show how a scaled-down motor imagery BCI-HIL application can be built. More specifically the application is a standard motor imagery session with a calibration phase and a feedback phase. Additionally, during the feedback phase, the resulting classification results are fed back to the stimuli program, altering the behavior, in order to showcase the more general HIL-application. While the performed experiment is simple, the example application still contains most major components of a human-in-the-loop BCI. The example could have been scaled down even further by, one step at a time, removing components such as the Admin GUI, classification-feedback, online calibration, and online signal processing. Removing all of the mentioned components would collapse the setup to a regular motor imagery data collection experiment. However, in the interest of generality, most system components are still included, while the experiment performed is chosen to be as simple as possible.

#### 4.1.1. Engine

The Engine is kept simple. During the calibration phase the program generates which motor imagery-tasks the subject will be asked to perform, and this command is passed to the Client GUI. When timestamps of the instructions being displayed on the monitor become available, the Engine creates the corresponding LSL event markers. In the feedback phase, the Engine is listening for the output results from the Calculate program, and sends them to the Client and Admin GUIs. Throughout the experiment, the Engine takes commands from the Admin GUI such as experiment metadata and when to switch between the calibration and feedback phases.

#### 4.1.2. Client and Admin GUI

In order to show only the bare minimum code and on-screen controls needed to run the experiment, both these programs are kept as simple as possible. The Admin GUI takes inputs which are forwarded to the Engine, while the Client GUI receives commands from the Engine saying what motor imagery commands and feedback to display to the subject.

#### 4.1.3. Calculate program

For this motor imagery experiment containing one calibration and one feedback session, we use the following Timeflux setup:

- **LSL graph:** Here, the LSL-streams of interest are the EEG data itself, the stream with markers indicating when stimuli are displayed (in this case instructions to the subject on what motor imagery to perform), as well as streams with high level communication such as signaling when to start/stop collecting data, train a ML-model, or when the training is done and the feedback phase can commence.
- **Preprocessing graph:** For preprocessing, two independent processing sequences are used in parallel: one for the calibration and one for the feedback session. Both sequences first apply a band-pass filter. The calibration sequence continues with the *Epoch* node (matching timestamps of stimuli markers with EEG-data in order to create epochs time-locked to the stimuli event). For a feedback session in a motor imagery experiment, there is no incentive to match epochs to stimuli events as the subject is intentionally encoding/modulating the mental state without being intrinsically time-locked to an external stimuli event. Therefore, the EEG data is cut into epochs with a fixed inter-epoch interval in a rolling window fashion. For this, the *Window* node is used. In the provided example code, the data is band-pass filtered between 8 and 30 Hz.
- **ML graph:** This graph consists of the *TrainingML* and *InferenceML* custom nodes. When the experiment starts, the epochs produced by the preprocessing graph are collected and saved to disk. Upon instruction from the Engine program, a scikit-learn pipeline model is trained on the available data and saved to disk. When ready, the InferenceML node takes over and loads the fitted model from disk and continuously classifies new EEG-epochs made available by the preprocessing graph.
  In the provided example code different scikit-learn pipelines are implemented. For instance, one of them calculates the covariance matrices for each epoch and uses minimum distance to mean classification on the Riemannian manifold. As emphasized above, any scikit-learn compatible classifier can be utilized by the researcher.

#### 4.1.4. Running a session

First, the computer needs to be setup to run Python programs, preferably using Python's virtual environments,^36^ Anaconda,^37^ or Miniconda.^38^ Additionally, a modern web browser has to be installed such as Google Chrome.^39^ In order to run a session, four separate programs need to be started: the Engine, the Client GUI, the Admin GUI, and the Calculate program.

To run the Engine, which is a Python program, a command line or terminal is used. Go to the engine folder using cd. The required Python modules are found in the requirements.txt file, and can for example be installed into your Python environment with the pip package installer using the command python -m pip install -r requirements.txt. Then, run the engine using the command python engine.py. Printouts and debug messages will be displayed in this command line window.

The Client GUI and Admin GUI are regular HTML web pages and runs directly in a web browser. To run these programs, find the admin and client folders respectively and then run admin.html and client.html either by opening the file-path in the web browser, or by clicking the files directly in the file system (assuming that a correct default application is set). Make sure that the Client GUI window is on the correct display when doing the latency calibration, as different screens will have different latencies in your setup.

The Calculate program is mostly running Python. However, since applications in Timeflux are defined and launched from yaml-files, the startup procedure is a bit different compared to when running the Engine. In order to run the BCI-HIL custom modules some extra setup is needed. For these instructions we refer to the README.md-file. Finally, to run the application from the command line, find the demo_MI/graphs/ folder. Here, launch the main yaml-file with the command timeflux main_demo_MI.yaml. Additional options can be specified with flags. For more info on these options use the command timeflux –help.

When the Engine and Calculate programs are run, they will start looking for LSL-streams on the local network. Make sure that the EEG hardware is powered on and configured to present itself as an LSL outlet. When the LSL-stream is found, a message will be written to the log output in the Engine's terminal window. Similarly, with debug messages activated, the Timeflux Receive nodes will also indicate when a matching LSL-stream has been found.

### 4.2. Clear by Mind BCI application

Clear by Mind is a game using the BCI-HIL framework presented in this paper. The game shows what a brain computer interface can do in a few minutes without any prior training, calibration effort or transfer learning in an unsupervised experiment. The aim of the demonstration is to raise interest in real-time reactive BCI research.

**Figure 6:**
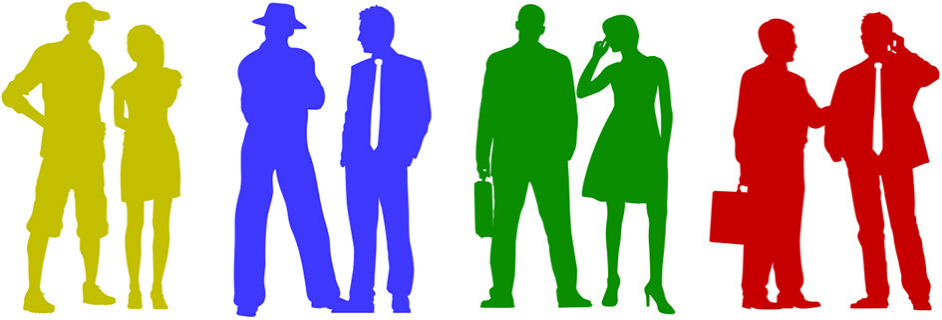
Visual stimuli used in the Clear by Mind game. The subject knows that one group of people, based on their color, is innocent. The subject is asked to count the number of times a person belonging to this group is displayed. Artwork: Kirsty Pargeter/Freepik.

The task in the game is for the subject to identify an innocent group of people that are incorrectly suspected in an ongoing investigation. This is done using a wireless EEG headset and a reactive BCI based on the oddball paradigm using the P300 response (Chapman and Bragdon, 1964). The subject has information about one group of people that are innocent, for example “the innocent people are green,” “yellow,” “blue,” or “red”. Examples of people from the different groups can be seen in Figure 6. In a series of rapidly displayed images, the subject will count the number of times a person belonging to the innocent category is shown, and the BCI will output probabilities of each category being the innocent.

One of the research questions that initiated the implementation of the Clear by Mind brain game is how to choose the stimuli sequence optimally. When using any event-related potential such as P300 in a reactive BCI, choosing a stimuli sequence algorithm that adapts to the classification results so far will outperform blind stimuli selection algorithms like pure randomness or round-robin algorithms (Tufvesson et al., 2023).

#### 4.2.1. Engine

Similar to the the Engine in the motor-imagery BCI application presented in Section 4.1 above, the Engine creates relevant event markers and acts as the mediator between the the Client and Admin GUI, and the Calculate program. Additionally, logic for deciding which stimuli to be displayed is implemented here.

#### 4.2.2. Admin GUI

The Admin GUI display is facing the audience and is not seen by the subject. In addition to accepting relevant operator inputs, it displays relevant information for the operator and audience such as the sequence of images shown and the current estimated probabilities output from the classification algorithm for each of the four different groups of suspects.

#### 4.2.3. Client GUI

The Client GUI display initially shows an attract mode slideshow and simple instructions for the subject to follow. During a trial, when requested by the Engine, the Client GUI shows the rapidly changing images that the subject either counts or ignores.

#### 4.2.4. Calculate program

The goal of the Calculate program in this case is to find the target class that the subject is focusing on, in an unsupervised fashion. The only information available in this setting is the raw EEG data, and which stimuli were displayed at different points in time.

- **LSL graph:** Similar to the motor imagery BCI application, the LSL-streams of interest are the EEG data itself, the stream with markers indicating when stimuli are displayed (in this case information on which stimuli were displayed), as well as the stream with general instruction regarding the experiment.
- **Preprocessing graph:** Contrary to the motor imagery BCI application, the *Epoch* node is used throughout the whole session for creating epochs. Only time-locked EEG-epochs matched to stimuli onset are used in this oddball paradigm.
- **Signal processing graph:** Since this is neither a regular supervised nor an unsupervised machine learning classification task, but rather a find the odd-one-out task, the previously mentioned *TrainingML* and *InferenceML* nodes are not used. Instead, a tailor-made node is used. Here, epochs are grouped corresponding to the color of the suspect being displayed when the epochs were collected. Any algorithm can then be applied to try to find the odd-one-out. In particular, an algorithm that averages epochs and compares pairwise distances between covariance matrices corresponding to the different classes is used.

#### 4.2.5. Running a session

**Figure 7:**
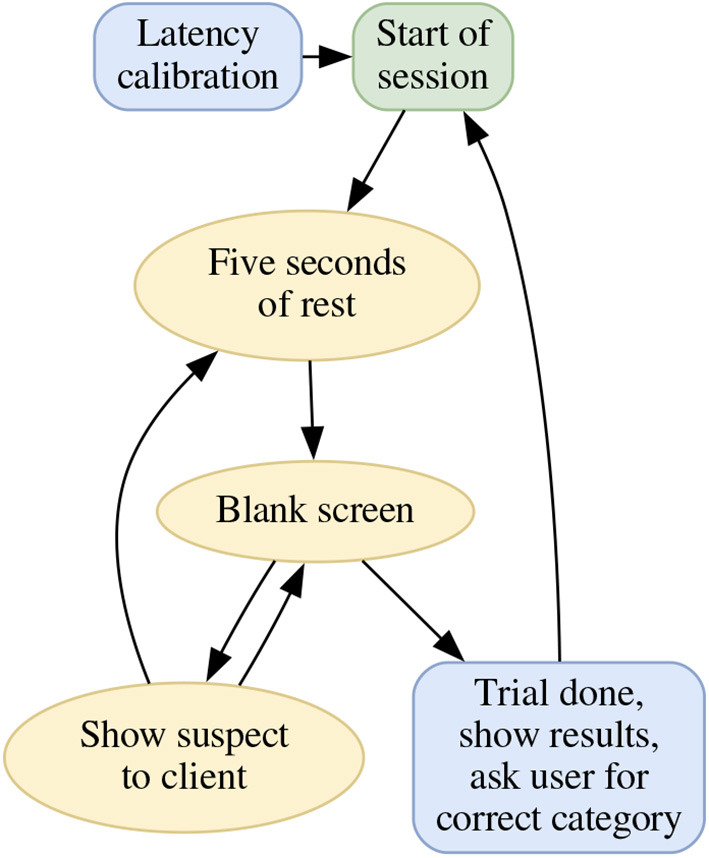
The flowchart for the Clear by Mind unsupervised classification experiment. At the start of the session, the subject is introduced to the task of counting suspects and gets instructions on what to expect during the experiment. Then, a five second rest state is used to let the subject focus, and then the Client GUI will show a number of suspects images in rapid succession. After a while the subject gets to rest again, allowing for a few seconds to relax, move freely and do eye blinks, before continuing counting another round of potential suspects. In this setup, the complete session lasts ~90 s. The latency calibration only needs to be done once, and is optional, but will improve the accuracy of the recorded marker's relative position to any saved EEG data when used for offline analysis.

To run the experiment, follow the steps in Section 4.1.4. When the EEG hardware and all four programs are up and running, an initial calibration phase (see Figure 7) is used to find the latency from display output to EEG input. This calibration should be done at least once per setup, since the latency depends on the specific combination of hardware.

### 4.3. Pitfalls and troubleshooting

There are numerous ways that the BCI-HIL research framework may or may not perform as intended. By carefully reading the log output of the Engine, most problems can be understood and corrected. Below is a list of potential configuration errors and how to handle them.

- **Engine debugging:** Read the console output from the Engine program. Debug messages useful for understanding many issues are printed here.
- **Client and Admin GUI debugging:** First, make sure to use the Google Chrome web browser for viewing the respective HTML files. The log output of these programs are found in the *Console* tab in the *Tools for Developers* sidebar. Debug messages useful for understanding many issues are printed here.
- **Timeflux and Calculate program debugging:** At runtime, Timeflux provide debug messages if the application is launched with the –debug flag. If things are not working as expected when building or customizing applications in Timeflux, a natural initial debugging step would be to verify that all data is passed as expected.
- **No LSL stream found:** If a wireless EEG hardware device is used, make sure that it is connected to the same wifi network as the computer that runs the Engine program. Also, make sure that this wifi network allows device-to-device direct communication with no firewall "protecting" devices from each other. This may be the case in corporate wifi setups. The solution is to setup your own local wifi network using a personal wifi router, or even running the experiment using a mobile hotspot from a smartphone. The availability of LSL-streams can also be checked by installing any LSL recorder software, and there make sure that the EEG hardware can be found.
- **EEG data loss or jitter:** The Client GUI in the Clear by Mind example brain game is setup to show EEG data with as low latency as possible. If frequent disruptions are noticed in the stream of incoming EEG data waveforms, the wireless setup might need to be optimized. To reduce jitter in the EEG stream, use wired communications wherever possible, and when forced to use wireless communication make sure that there are as few disturbing devices using the same frequency bands as possible. Regarding Bluetooth, it is a good idea to turn off other Bluetooth devices in closer proximity than 30 m. Regarding wifi, a wifi analyzer app on a smartphone can be used to scan for and identify other wifi nets and routers that may introduce congestion and impair the wireless channel. It is also possible trying to switch to another wifi channel in the router providing the experiment wifi.
- **LSL timestamp units:** Beware that EEG hardware using LSL can have their own interpretation on how to produce timestamps, especially when it comes to the unit: seconds, milliseconds, or nanoseconds. The timestamp may also be offset with zero being the boot time of the system, the Unix epoch in 1970 or any other arbitrary offset.
- **Cloud computing:** In this paper, we intentionally refrain from referring to any particular commercial cloud services or providers, and consider “cloud computing” as any remote computer outside of your local network. Cloud computing services can provide you with virtual machines that support the websocket technology that we use as communication channel between modules in BCI-HIL. The deployment, security, and management of cloud-native technology is beyond the scope of this method paper.

### 4.4. Experiment preparations

Before the BCI-HIL framework is used some preparations are needed.

#### 4.4.1. Human-in-the-loop latency calibration

At least once for every unique combination of computer, display, loudspeaker, headphones, EEG hardware, and network connection, you should estimate the latency between stimuli and EEG signal. The BCI-HIL research framework attempts to keep the jitter in this latency as small as possible. The average value of the latency is unknown, but often below half a second, which depending on circumstances can be regarded as small or large.

#### 4.4.2. Electrode impedance

Preparing the EEG headset is an art in itself. Some EEG hardware has the possibility to directly measure the electrode impedance guiding the application of wet abrasive gel to minimize the artifacts that will arise from high impedance electrode to skin coupling, as described by Browne (1957).

When using simpler EEG hardware, one way of detecting less-than-ideal impedance is to watch for the artifacts directly. EEG is measuring signals in the range of millivolts, and the electromagnetic environment of today contains a lot of noise sources that will interfere with the measurements. In almost any location where EEG measurements are done, there will be 50 or 60 Hz disturbances coming from the electricity distribution system in walls, floors, and ceilings. We can use these artifacts to roughly estimate if an electrode has a low enough impedance between the electrode and the skin, since whenever the impedance gets high, the 50/60 Hz amplitude will rise. A narrow band-pass filter around 50 Hz can be added to measure the energy in the signal, and then provide visual feedback on the Admin and Client GUI displays for all the measured EEG channels. The artifact amplitudes could then assist in aligning the electrodes and improve their connectivity. Since non-artifact EEG signals are inherently low amplitude, implementing a 1 Hz high pass filter should also get a good enough power estimation for EEG electrode adjustment guidance.

A filter should be used to reduce these high impedance artifacts' impact on your online analysis. This could either be a low pass filter, or a 50 or 60 Hz notch filter. Do note that any kind of online filtering needs to make a proper trade-off between frequency and phase response vs. non-causality. Only introduce a filter if you know it makes sense to use it.

## 5. Discussion

### 5.1. Considerations when choosing a BCI research framework

Having read up to this point, you have attained a substantial level of understanding concerning the research methodology and the requisite tools for BCI systems. Selecting a BCI framework requires a comprehensive examination of the framework's intended purpose, as well as its target users and implementation methods. We have outlined a list of desirable attributes for a BCI research framework in Section 3.1.

For example, if you are a scientist interested in experimenting with and developing new algorithms for BCIs or data analysis, you will probably also be proficient in programming. Such a user will most likely be interested in open-source code, a high level of customizability, and modularity (in the sense that different components of the system can be exchanged by others) which our framework BCI-HIL provides. Having a GUI and ready-to-use modules might be of relatively low importance in this case.

For researchers less proficient in coding, open-source code and complete customizability might be less important, while interaction with a large set of easily combined standard components and algorithms is valued highly. If you remain uncertain regarding the selection of an appropriate BCI research framework, we suggest opting for a Graphical User Interface (GUI) based drag-and-drop configuration, such as those presented in Section 2.2.1.

### 5.2. Considerations when planning your experiment

#### 5.2.1. Ethical aspects

It is imperative for any experimental study to undergo an ethical review by an external committee. For instance, in accordance with the regulations outlined by the European General Data Protection Regulation (GDPR), EEG data is regarded as personal data when it includes information about an individual's physiology, health, or mental states. Although these properties are not typically utilized in a BCI setting, they are still inherent in the underlying EEG signal. As a result, it is essential to consider brain data to be equally sensitive as medical data and to treat it accordingly. One solution to ensure data privacy is to ensure that stored EEG data is kept separate from any personal identifiers. Specifically, any cloud computing devices responsible for processing the EEG data should not handle any metadata that could potentially be utilized to link the data to an identifiable individual. By implementing this approach, the EEG data can be appropriately treated as pseudonymized. For a thorough discussion about EEG signals and data privacy, see the article by Rainey et al. (2020).

Regardless, your experiment should include a consent form, which subjects will need to sign before having their data recorded and used.

#### 5.2.2. Eye blink removal

Depending on your experimental setup, there will be a certain amount of subject induced artifacts in the measured EEG data. These are unwanted segments where noise might completely mask out or deteriorate the signal-to-noise ratio of the EEG signal. Thus, parts of the time series could be unusable. One way of dealing with these artifacts is trying to limit them or control when they happen. Asking subjects to refrain from movements during parts of the experiment will lower the amount of noise due to mechanical or muscle movement. It also possible to introduce eye blink pauses in the experiments, trying to keep the amount of usable EEG data high. Another common practice is to add fixation crosses for the subject to focus on, to reduce the number of saccades. If you cannot avoid getting artifacts into your EEG signals, Jiang et al. (2019) gives an overview of approaches to EEG artifact removal.

#### 5.2.3. Baselining

The EEG signal quality can be improved by using baselining, which means that the signals are reset to a starting level at the onset of an event, canceling out drifting potentials between the EEG electrodes. Baselining is easier to use than high-pass filters which are known to deform relevant parts of the EEG-waveform in for example event-related potentials. Additionally, in contrast to any practically useful casual high-pass filter, baselining does not need to add processing delay to the EEG signal, since the correction is based on data that has already been acquired.

#### 5.2.4. Algorithm optimization with simulated EEG

Even though all EEG-signals and stimuli are recorded, there is a point where changing the algorithms also would have changed the response or behavior of the subject. To be able to experiment with algorithms offline in this setting, one would need to simulate a model of human behavior. There are models for generating EEG data on every level, from individual neurons up to single EEG scalp electrode ERP responses, as described in the book by Ermentrout and Terman (2010). Naturally, these simulation models are simplifications compared to a real human brain and will only to some extent help when optimizing algorithms offline. However, offline processing can help in finding artifacts as well as improve the understanding of the signals and noise present in the current experiment.

#### 5.2.5. Gradual improvements and iterations

To optimize performance in every unique BCI situation one should plan for making many iterations. Every paradigm, subject and specific setup is going to be at least slightly different. Additionally, insights gained from offline analysis might lead to changes in the setup that help to improve the performance of online human-in-the-loop system. But of course, the effect of these changes will only be seen when running yet another iteration of the online system, as illustrated in Figure 2.

## 6. Conclusion

In this paper, we have presented an open-source BCI research framework for the next generation of brain-computer interfaces, addressing the challenge of fast prototyping for online classification of neural activity. We introduced the BCI-HIL (see text footnote 35) framework for real-time classification, analysis, and computations to bring the human into the loop of learning, evaluation, and improvement. This approach can lead to shorter calibration times and the possibility of researching new ideas and expanding where and when brain-computer interfaces can be used.

## Data availability statement

Code for the framework is publicly available at https://www.bci.lu.se/bci-hil (or at https://github.com/bci-hil/bci-hil).

## Author contributions

MGN: conceptualization, methodology, writing—original draft, writing—editing, software, investigation, and project administration. PT: conceptualization, methodology, writing—original draft, writing—editing, visualization, and investigation. FH: software, investigation, and writing—review. MJ: supervision and writing—review and editing. All authors gave final approval for publication and agreed to be held accountable for the work performed therein.

## Footnotes

^1^ spdx.org/licenses

^2^ gnu.org/licenses

^3^ sccn.ucsd.edu/wiki/BCILAB

^4^ sccn.ucsd.edu/eeglab

^5^ fieldtriptoolbox.org

^6^ bci2000.org

^7^ openvibe.inria.fr

^8^ bitbucket.org/kloostermannerflab

^9^ github.com/gumpy-bci/gumpy

^10^ timeflux.io

^11^ sccn.ucsd.edu/eeglab

^12^ mne.tools

^13^ bbci.de/pyff/index.html

^14^ psychopy.org

^15^ mbraintrain.com

^16^ choosemuse.com

^17^ neurosity.co

^18^ mathworks.com

^19^ python.org

^20^ julialang.org

^21^ javascript.com

^22^ octoverse.github.com/2022/top-programming-languages

^23^ html.spec.whatwg.org

^24^ w3.org/Style/CSS

^25^ labstreaminglayer.org

^26^ labstreaminglayer.readthedocs.io/info/intro.html

^27^ github.com/sccn/xdf

^28^ nodejs.org

^29^ timeflux.io

^30^ zeromq.org

^31^ superpowered.com/webbrowserlatency

^32^ leobodnar.com

^33^ displaylag.com/display-database

^34^ rtings.com/tv/tests/inputs/input-lag

^35^bci.lu.se/bci-hil (or at: github.com/bci-hil/bci-hil).

^36^ docs.python.org/3/library/venv.html

^37^ anaconda.org

^38^ docs.conda.io/en/main/miniconda.html

^39^ google.com/chrome
